# Supplementary material for: HIV-1 Nef Protein Affects Cytokine and Extracellular Vesicles Production in the GEN2.2 Plasmacytoid Dendritic Cell Line
Source: Viruses. 2021 Dec 31;14(1):74. doi: 10.3390/v14010074 (PMC8780779; doi:10.3390/v14010074)
Supplement: Supplementary file 1 [file viruses-14-00074-s001.zip › viruses-1486389-supplementary.pdf]

Supplementary table

Table S1. Antibodies reagents for FC analysis

Cells	Antibodies	Producers
**PBMCs**	Lineage-FITC/BDCA2-PE/ HLADR-PerCP/CD123-APC CD3-FITC/CD19-PE/CD14-APC	BD Biosciences; MACS Miltenyi; BD Biosciences; eBioscience; BD Biosciences; BD Biosciences; BD Biosciences.
**PBMCs – pDCs**	Lineage-FITC/BDCA2-PE/ HLADR-PerCP/CD123-APC	BD Biosciences; MACS Miltenyi; BD Biosciences; eBioscience.
**PBMCs – Monocytes (PBLs)**	CD3-FITC/CD19-PE/CD14-APC	BD Biosciences; BD Biosciences; BD Biosciences
**PBMCs – Monocytes – pDCs (PBLs – pDCs)**	CD3-FITC/CD19-PE/CD14-APC	All purchased from BD Biosciences
**Monocytes**	CD3-FITC/CD19-PE/CD14-APC	All purchased from BD Biosciences
**pDCs**	CD19-FITC/CD123-PE/CD3-APC	BD Biosciences; eBioscience; BD Biosciences

Supplementary figures

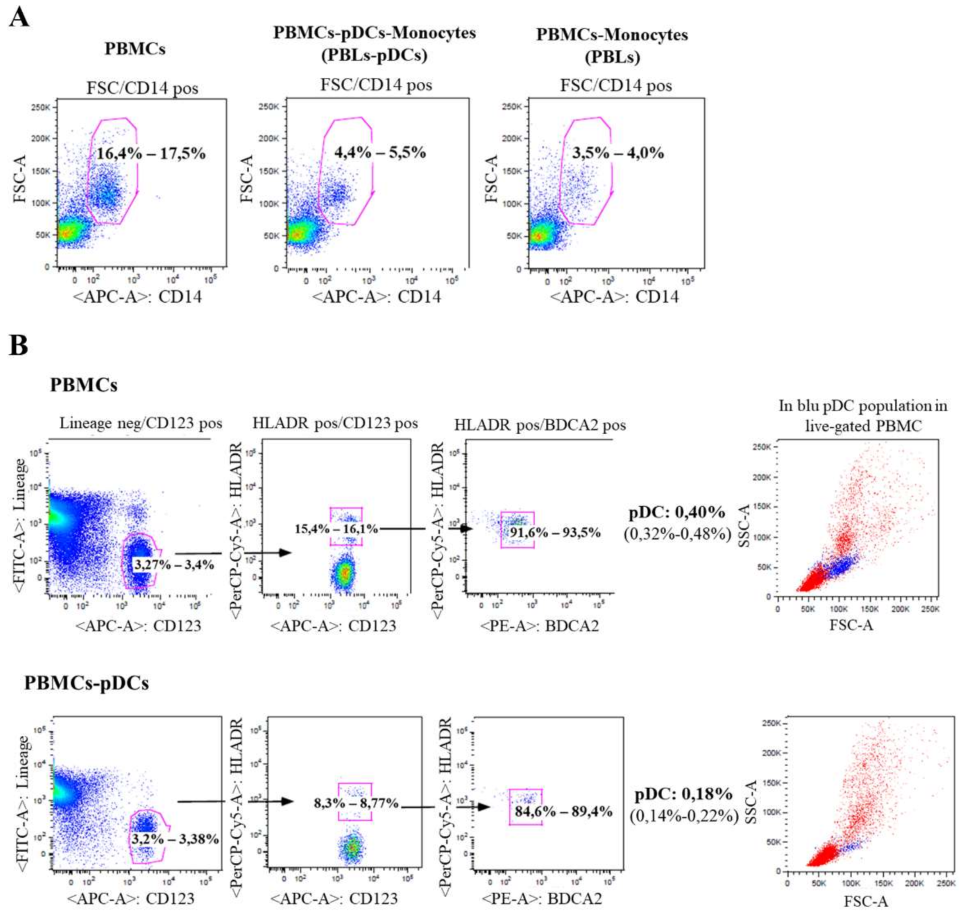

Figure S1. Purity of isolated cells. Dot plots show the forward light scatter/SSC profile of the cells. (A) Monocyte depletion analysis. The frequency of monocytes was determined within PBLs and PBLs-pDCs as cells positive for CD14. (B) pDC depletion analysis. The frequency of pDCs was determined as cells positive for CD123 and BDCA2 (CD303) within peripheral blood mononuclear cells (PBMCs).

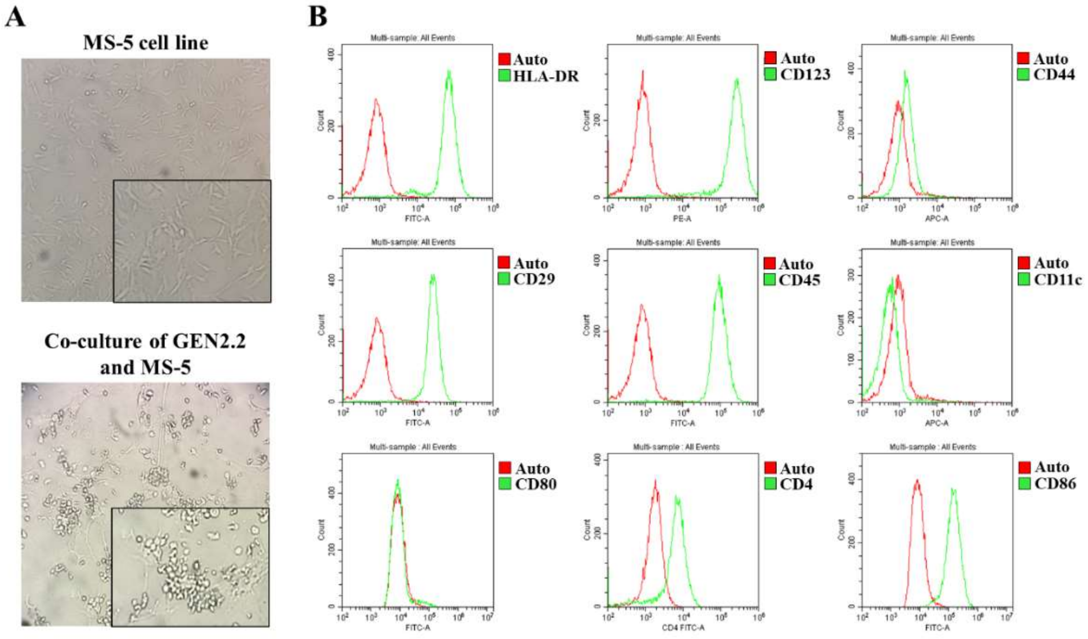

Figure S2. Morphology and phenotype of GEN2.2 cell line. (A) Representative images of MS-5 stromal cell line used as feeder layer (upper) and of GEN2.2 during the culture on irradiated MS-5 cells (bottom). At the bottom right of both images, a magnified detail is shown. (B) Phenotypic characteristics of fresh GEN2.2 cells analyzed by flow cytometry. For surface staining, 0.3 x 10^6 cells were harvested from the culture and processed as reported in Materials and Methods section. The autofluorescence of the cells is indicated in red, whereas the expression of the specific markers in green. A representative analysis, out of three independent analyses that yielded similar results, is shown.
